# Coating Extrusion Characteristics of Thin-Walled Tubes for Catheters Using Thermoplastic Elastomer

**DOI:** 10.3390/polym17010102

**Published:** 2025-01-02

**Authors:** You He, Huanlao Liu, Yulin Wang, Weikang Hu

**Affiliations:** Guangdong Engineering Technology Research Center of Small Household Appliances Innovation Design and Manufacturing, School of Mechanical Engineering, Guangdong Ocean University, Zhanjiang 524088, China; 2112205034@stu.gdou.edu.cn (Y.H.); yu-linwang@163.com (Y.W.); huweikangs@163.com (W.H.)

**Keywords:** wire coating extrusion, non-isothermal, without shaping section, drag velocity, inlet flow rate, die temperature

## Abstract

During the production of medical thin-walled tubes, a thin coating layer is required. This requirement reduces the cross-sectional clearance area of the straight section flow channel formed by the mandrel and the die, leading to excessive pressure of the polymer melt at the shaping section, elevated die pressure, and backflow of the material melt, all of which directly impact the quality of the coating layer. To address these issues, this study conducted a non-isothermal numerical simulation of coating models both with and without a shaping section. It analyzed the impact of the coating model without a shaping section on the coating layer’s thickness and the stability of the coating flow field under varying drag velocities, inlet flow rates, and die temperatures. Furthermore, it compared these results with those of coating extrusion using shaping section runners and investigates how different flow channel parameters affect the extrusion characteristics of the coating layer. The results showed that the setting of the shaping section could not eliminate the extrusion expansion in wrap extrusion. In comparison to coating extrusion utilizing a shaping section die, the process without a shaping section die reduced die pressure drop by 35% to 40%, decreased energy consumption, and enhanced the quality of the coating layer. Finally, an amorphous segment-coating extrusion die was designed based on the simulation results, and coating extrusion experiments were carried out using the designed and fabricated die.

## 1. Introduction

The demand for endoscopes is rising due to the global aging population and increasing environmental concerns. Endoscopic technology is widely recognized by clinicians and patients for its diagnostic and therapeutic efficacy, minimally invasive nature, low infection risk, and advantages in rapid postoperative recovery. These benefits have made endoscopic products one of the fastest-growing innovations in the medical device industry, gradually transforming modern medical diagnosis and treatment [1]. As demand for endoscopic insertion tubes in the interventional field grows, a coating layer formed through the wire coating extrusion process is increasingly utilized in these tubes. The quality of this coating layer is gaining attention as a critical factor influencing the tubes’ medical performance. Currently, domestic medical endoscope insertion tubes often exhibit significant defects in the coating layer, including uneven appearance, rough surface texture, and inconsistent coating thickness. These defects can lead to wear or breakage of the coating layer during clinical use, compromising the overall performance and safety of the endoscopic insertion tubes. Consequently, improving the quality of the coating layer in the production of medical endoscopic insertion tubes has emerged as both a technological challenge and a primary research focus in coating extrusion [2,3].

Scholars, both domestically and internationally, have extensively researched the coating extrusion of sheets, tubes, profiles, and microtubes. Wagner and S. Gouran [4] employed a least-squares approach to solve nonlinear differential equations, aiming to predict the velocities and temperatures of non-Newtonian fluids in pressure molds. P. Poungthong et al. [5] applied the method of Jones (1964) to determine the velocity and thickness distribution of the cladding. They then integrated this distribution to calculate the flow rate and, consequently, the mean thickness. Zhang et al. [6] utilized numerical simulation to elucidate the intrinsic relationship between flow balance coefficients, rheological parameters, and the exit cross-sectional geometry of extruded thin-walled profiles. M.A. Javed [7] performed a non-isothermal study of the wire coating process utilizing the Carreau–Yasuda fluid model. This study explored the relationship between model parameters and factors such as wire radius, volumetric flow rate, and the tractive force necessary for wire pulling. Chen et al. [8] proposed four methods for optimizing the design of coating mold runners through structural analysis of profiles. Yin et al. [9] utilized Polyflow to examine how process parameters in conventional and gas-assisted wrap extrusion affect the pressure, velocity, and shear stress fields. S. Cho et al. [10] investigated the effect of extrusion process parameters such as screw speed (inlet flow rate), haul-off speed, and air injection pressure on the ovality and inner and outer diameter dimensions of thin-walled tubes. Ahmad et al. [11] examined the effect of four process parameter variables on the tensile strength of polylactic acid (PLA) samples. Ahmad et al. [12] derived von Mises plasticity material model and boundary conditions that accurately represented the behavior of the specimen under uniaxial tension load, with only minimal differences between actual and theoretical results. Zhao et al. [13] investigated the effects of primary structural parameters of the mold runners on extrusion flow uniformity. Based on the identified optimal runner structural parameters, a dual-lumen microtube extrusion die was designed and fabricated. The results of the extrusion experiments confirmed the accuracy and effectiveness of the method. Shah et al. [14] solved the constitutive and energy equations of motion for velocity, pressure distribution along the radial direction, and temperature distribution using perturbation theory. Zeeshan et al. [15] utilized a pressurized coating die to investigate the use of PTT fluid as a polymer material for wire in a magnetic field. Exact solutions were derived for the flow field, flow rate, temperature profile, thickness of the wire coating, volume flow rate, and shear stress. G.B. Jin et al. [16], using polypropylene as the extrusion material, experimentally investigated the effect of extrusion process parameters on the shape accuracy of the five-lumen microtubes, which was evaluated by the ellipticity of the microtubes profile.

Previous research on coating extrusion primarily focused on cables, which possess a thicker coating layer, thus eliminating the need to consider die pressure during actual production [17]. However, when coating thin-walled braided medical tubing in production, the requirement for a thin coating layer results in a reduced cross-sectional clearance area in the shaping section of the runner formed by the mandrel and the die. In this scenario, the shaping section of the mold can cause excessive pressure on the molten material, leading to backflow. This backflow can result in fluctuations in the coating layer’s thickness, instability in the coating production process, and poor appearance of the coating. For pseudoplastic fluids, temperature influences the viscosity of the melt, thereby altering its flow rate distribution and pressure distribution, which in turn affects the quality of the coating layer. Therefore, the effect of die temperature during the wire coating extrusion process should not be ignored [18].

This paper employs Polyflow (2021R1) finite element analysis to simulate a coating layer model with an inner diameter of 5.4 mm and an outer diameter of 6.2 mm under non-isothermal conditions. The study analyzes how process and runner parameters influence the size of the coating layer and the stability of the flow field. Based on the simulation results, an amorphous segment-coating extrusion die was developed. Coating extrusion experiments were subsequently conducted using this die. This research enriches the theory of thin-walled pipe coating extrusion preparation and provides a reference for formulating process parameters in actual production.

## 2. Experimental Methods

### 2.1. Material Rheology Experiment

**Figure 1:**
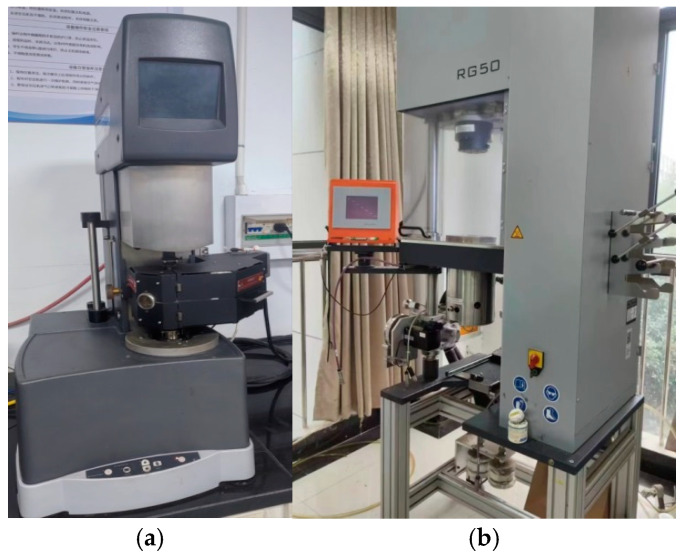
Rheological test equipment. (**a**) ARES-G2 (TA Instruments, USA) rotational rheometer; (**b**) RG50 (Gottfert, Germany) capillary rheometer.

The coating material used in this study is extrusion-grade thermoplastic polyurethane (TPU). Some physical parameters of the material are listed in Table 1, while the experimental equipment is depicted in Figure 1. Due to the high water absorption rate of TPU, it must be dried before testing to prevent errors from water evaporation. The polymer was dried in an electric blast drying oven at 80 °C for 4 h and then molded using a plate vulcanized into a circular specimen with a diameter of 25 mm and a thickness of 2 mm [19]. The hot-pressing temperature was set at 180 °C, with a maximum pressure of 15 MPa, to ensure that the specimen exhibited uniform wall thickness, was free of bubbles, and was suitable for use in a rotational rheometer. The rheological data for shear rates ranging from 0.01 s^−1^ to 1000 s^−1^ were measured using a rotational rheometer (TA Instruments ARES-G2, New Castle, DE, USA) at temperatures of 170 °C, 180 °C, 190 °C, and 200 °C, while the rheological data for shear rates ranging from 1000 s^−1^ to 5000 s^−1^ were measured using a capillary rheometer (Gottfert-RG50, Buchen, Germany). The capillary rheometer was tested using four orifice aspect ratios (10/1, 20/1, 30/1, and 20/2) and calibrated for zero shear, inlet pressure drop, and wall slip to obtain viscosity data.

**Figure 2:**
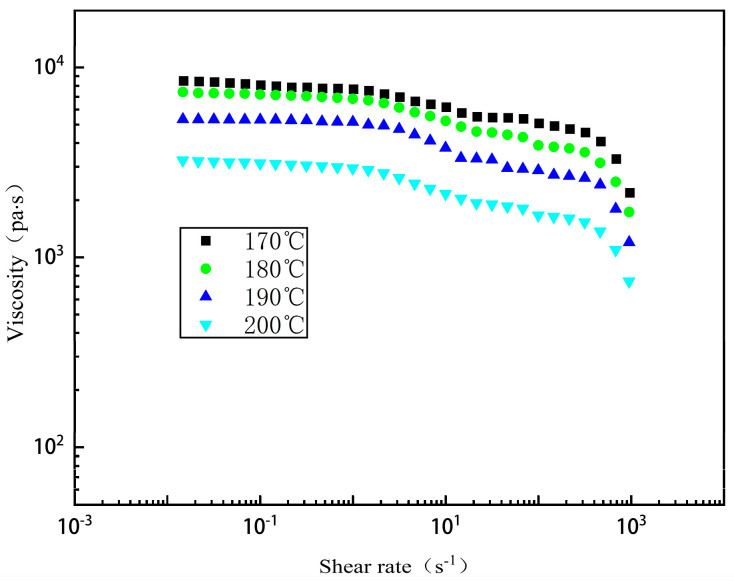
Variation in viscosity according to shear rate (TPU ESTANE-58271).

Figure 2 shows a plot of the steady-state rheological data measured using both the rotational and capillary rheometers at 170 °C, 180 °C, 190 °C, and 200 °C. The figure shows that at lower shear rates, the apparent viscosity varies minimally with shear rate, maintaining a nearly constant zero-shear viscosity value. As the shear rate increases to 0.1 s^−1^, the molten material exhibits a clear shear thinning phenomenon. The movement of polymer chain segments intensifies, reducing entanglement, and the apparent viscosity decreases significantly with the shear rate [20,21]. From the steady-state rheological test data plots, it is evident that the Brid–Carreau model fits the material’s rheological data well. Particularly at low shear rates, when the shear rate is less than 10 s^−1^, the shear thinning effect of the TPU melt is not pronounced, and the Brid–Carreau model accurately captures this behavior.

After several fitting attempts, the Brid–Carreau–Arrhenius model was selected as the mathematical model for non-isothermal generalized Newtonian fluid in this study. The expression for its viscosity is provided in Equation (3).

(1) $$\eta_{a}=\eta_{\infty }+(\eta_{0}-\eta_{\infty }){\left[1+(\lambda \dot{\gamma }{)}^{2}\right]}^{(n-1)/2}$$

(2) $$H\left(T\right)=exp[\alpha (\frac{1}{T-T_{0}}-\frac{1}{T_{a}-T_{0}})]$$

(3) $$\eta \left(T\right)=\eta_{a}\times H\left(T\right)$$

where $\eta_{a}$ is the apparent viscosity of the polymer melt; $H\left(T\right)$ is a function of temperature; $\eta_{0}$ is the zero-shear viscosity; $\eta_{\infty }$ is the infinite shear viscosity; λ is the melt relaxation time; $\dot{\gamma }$ is the shear rate; n is the non-Newtonian exponent of the melt, dimensionless; α is the temperature coefficient; T is the unknown thermodynamic temperature; $T_{0}$ is the reference temperature; and $T_{a}$ is the reference temperature at $H\left(T\right)=1$.

The individual parameters of the mathematical model used for simulation are shown in Table 2.

### 2.2. Finite Element Modeling

**Figure 3:**
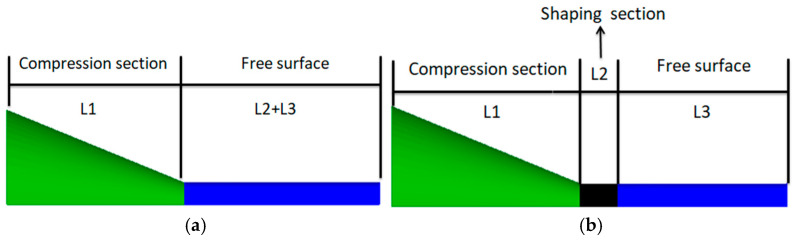
Physical model of the coating extrusion (**a**) without shaping section coating extrusion; (**b**) with shaping section coating extrusion.

**Figure 4:**
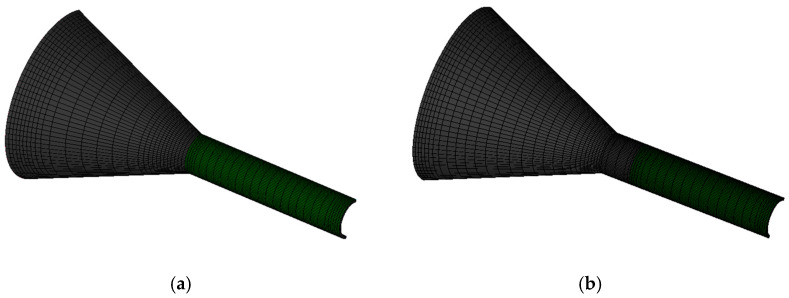
Meshing of the melt of the numerical analysis (**a**) without shaping section coating extrusion; (**b**) with shaping section coating extrusion.

The finite element model of the coating layer consists of three parts: a compression section, a shaping section, and a free-melt extrusion section. Figure 3a illustrates the coating model that excludes the shaping section, whereas Figure 3b illustrates the model that includes the shaping section. Due to the symmetry of both models with respect to the central axis of the cladding mold, only one-half of the models are utilized for finite element simulation. This is demonstrated in Figure 3, the green color is the compression section, the black color is the shaping section, and the blue color is the free-melt extrusion section. The dimensions of the model are presented in Table 3. To improve the accuracy of the results, appropriate mesh refinement is applied to critical areas that influence melt flow, as illustrated in Figure 4 [22].

#### 2.2.1. Engineering Assumptions

(1) The melt flow is a fully developed steady-state laminar flow with no time-dependent variables in the equation. (2) The heat transfer and heat diffusion of the melt are constant, with a constant specific heat capacity and heat transfer coefficient. (3) The melt is an incompressible non-Newtonian steady-state laminar flow. (4) The TPU melt is highly viscous, making the effects of inertial and gravitational forces negligible. (5) There is no slippage on the wall surfaces of the flow channel.

#### 2.2.2. Boundary Condition

(1) Inlet boundary conditions: It is assumed that the inlet melt is a fully developed flow, satisfying condition $\partial v_{y}/\partial z=0,v_{x}=v_{z}=0$, where $v_{y}$ is the flow rate of the melt along the extrusion direction. The melt inlet flow rate is set to $Q=400\;{mm}^{3}/s$. For the temperature boundary conditions, the inlet temperature of the wrapping mouth mold is set to a fixed temperature T=443.15 K. (2) WaLL1 boundary conditions: For the dynamic boundary, the melt wall satisfies the condition $V_{n}=V_{s}=0$. For the temperature boundary condition, the temperature of the wall of the cladding mouth mold is set to a fixed temperature of T=453.15 K. (3) Free surface boundary conditions: For the dynamic boundary, the melt free surface satisfies the condition $f_{n}=0,\;f_{s}=0\;\mathrm{and}\;v_{n}=0$. For the temperature boundary conditions, since the free boundary contacts the outside environment, resulting in convective heat exchange, the temperature boundary is set based on heat flux density conditions. Only convective heat transfer is considered, while thermal radiation is ignored. The mathematical expression used is $q=-k\nabla T=h\left(T-T_{\mathrm{air}\;}\right)$, where 10 $W/(m^{2}\cdot K)$ is the value for the thermal conductivity coefficient, h is the natural convection coefficient of air, T is the melt temperature, and $T_{\mathrm{air}\;}$ is the outside air temperature, set at 300.15 K. (4) WaLL2 melt and braided tube composite interface boundary conditions: For the dynamics boundary, the braided tube traction speed is $v_{c}=20\;mm/s$, with traction only along the extrusion direction. Thus, the velocity is imposed along the *y*-axis in the Cartesian coordinate system, which corresponds to the melt extrusion direction. The traction velocity $v_{c}$ is applied (Cartesian velocities imposed ($v_{x}=0$, $v_{y}=v_{c}$, $v_{z}=0$)). For the temperature boundary, the temperature is set to a fixed value of T=453.15 K. (5) Exit boundary conditions: For the kinetic boundary, although polymer microtube extrusion typically requires a certain traction force, in the simulation of wrap extrusion, the melt is subjected to the traction of the braided tube. Therefore, the velocity is set to $f_{n}=0,v_{n}=0,v_{s}=0$. For the temperature boundary, since the temperature of the flow exiting the mold is unknown, it is set to the exit (outflow) temperature conditions. (6) Axisymmetric boundary conditions: For the dynamics boundary, the symmetric surface satisfies the condition $f_{s}=v_{n}=0$. For the temperature boundary, since the temperature at the interface is continuous, the symmetric temperature condition is satisfied.

**Figure 5:**
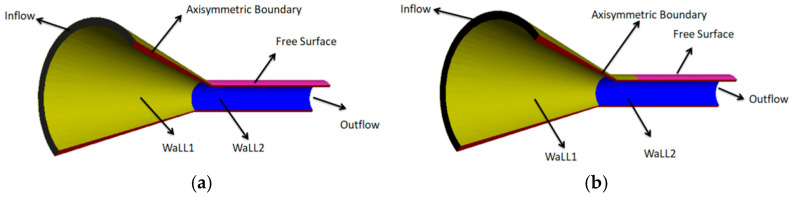
Boundary conditions of the numerical analysis (**a**) without shaping section coating extrusion; (**b**) with shaping section coating extrusion.

The specific boundary settings are shown in Figure 5.

## 3. Results and Discussions

### 3.1. Velocity and Pressure Field Analysis

**Figure 6:**
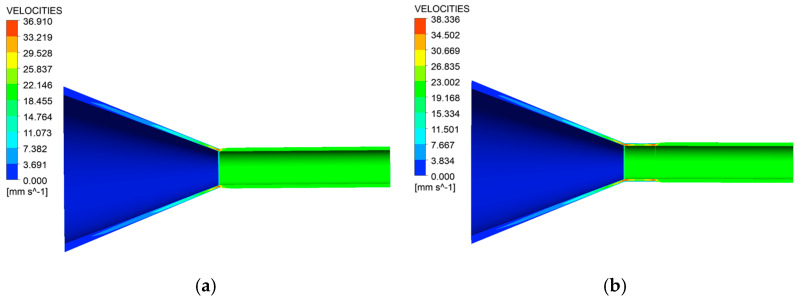
Distribution of the cloud charts of velocity (**a**) without shaping section coating extrusion; (**b**) with shaping coating extrusion.

With the current boundary condition settings, the velocity distribution clouds along the extrusion direction for the two wrapping head models are shown in Figure 6. In this figure, (a) represents the wrapping extrusion without a shaping section, and (b) represents the wrapping extrusion with a shaping section. From the figure, it is observed that the flow rate of the melt gradually increases as the cross-section of the runner decreases in the compression section, with the maximum flow rate occurring near the outlet of the coating mouth mold. At the end of the over mold, the melt flow rate experiences a significant sudden change. As the melt passes through the die and flows out for a certain distance, its flow rate gradually stabilizes [23]. It eventually reaches and maintains a constant flow rate that matches the traction speed of the braided tube, allowing it to flow smoothly along the extrusion direction. Under identical process parameters, comparative analysis indicates that the extrusion swelling effect of wrap extrusion, whether with or without a shaping section, appears nearly identical. This contrasts with conventional extrusion, where including a shaping section significantly reduces or eliminates extrusion swelling. The extrusion expansion phenomenon in conventional extrusion occurs partly because the elastic energy from the orientation and stretching of the molecular chains seeks to return to equilibrium once the polymer is released from the mold. Additionally, it results from the redistribution of the flow rate after exiting the mold, transitioning from the gradient of laminar flow to a rapid transformation into blocking flow. For coating extrusion, the melt experiences continuous traction from the internal tubing after exiting the die, regardless of the presence of a shaping section. This traction interferes with the orientational stretching of the molecular chains, prompting the melt to swiftly transition from a gradient laminar flow to a drag flow, instead of a blocking flow, after leaving the die [24,25]. As illustrated in Figure 6a,b, beyond the orifice die, the melt velocity is entirely governed by the pipe drag velocity, eventually stabilizing. Consequently, at the exit of the compression section, there is no significant difference in the outer diameter of the cladding layer produced by cladding extrusion, whether or not a shaping section is used.

**Figure 7:**
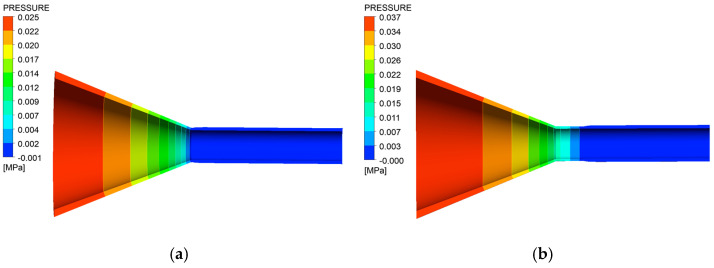
Distribution of cloud charts of pressure (**a**) without shaping section coating extrusion; (**b**) with shaping coating extrusion.

Figure 7 shows the pressure distribution obtained from the simulation. From the figure, it can be observed that the high viscosity of the TPU melt results in a large inlet pressure at the melt entrance. As the melt enters the compression section of the die, the cross-sectional area of the flow channel gradually reduces towards the exit of the compression section. Consequently, the pressure exerted on the melt decreases along the extrusion direction. When the melt flows out of the wrapping die without a shaping section, the pressure tends to stabilize. Conversely, in the case of wrapping extrusion with a shaping section, the pressure decreases as the melt enters the shaping section and then stabilizes once the melt has completely exited the shaping section.

### 3.2. Analysis of Change in Outer Diameter of Coating Layer

The primary process parameters in the medical thin-walled braided tube coating extrusion process include the haul-off speed of the braided tube, the melt inlet flow rate, and the orifice temperature. The study of the overmold dimensions encompasses the length of the shaping section, the length of the compression section, and the thickness of the overmold. The thickness is calculated based on the outer diameter of the braided tube and the inner diameter of the overmold. In this section, numerical simulations are employed to examine how these parameters affect the quality of the cladding layer during the extrusion process. This analysis lays the groundwork for the subsequent design and refinement of the coating extrusion die. To systematically analyze the effects of process and runner parameters on overmolding extrusion, only specific parameter values are varied according to the analytical objective, while all other boundary conditions remain constant (see Table 4). In analyzing the effect of braided tube haul-off speed on the coating extrusion process, the traction speed of the braided tube was set to $v_{c}=15\;mm/s$, $v_{c}=20\;mm/s$, $v_{c}=25\;mm/s$, $v_{c}=30\;mm/s$, $v_{c}=35\;mm/s$, $v_{c}=40\;mm/s$; when analyzing the effect of melt inlet flow rate on the coating extrusion process, the melt inlet flow rate was set to $Q=250\;{mm}^{3}/s$, $Q=300\;{mm}^{3}/s$, $Q=350\;{mm}^{3}/s$, $Q=400\;{mm}^{3}/s$, $Q=450\;{mm}^{3}/s$, $Q=500\;{mm}^{3}/s$; and when analyzing the effect of die temperature on the coating extrusion process, the die temperature was set to T=443.15 K, T=453.15 K, T=463.15 K, T=473.15 K. Finite element analysis was conducted on both the stereotyped and amorphous section models. In the study of how the shaping section length affects the extrusion quality of the braided pipe covering layer, the shaping section length $L_{1}$ was set to 0 mm, 5 mm, 10 mm, 15 mm, and 20 mm. For examining the impact of the compression section length, $L_{2}$ was set to 45 mm, 47.5 mm, 50 mm, 52.5 mm, and 55 mm. In studying the effect of wrapping thickness on extrusion quality, the wrapping thickness $t_{1}$ was set to 0.6 mm, 0.7 mm, 0.8 mm, 0.9 mm, and 1.0 mm.

**Figure 8:**
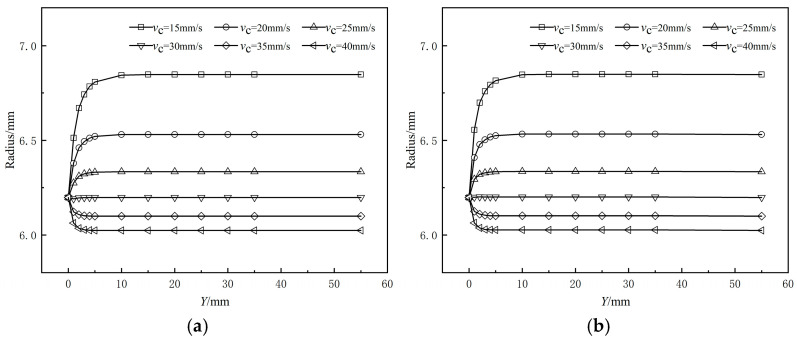
Effects of the drag velocity on the coating radius (**a**) without shaping section coating extrusion; (**b**) with shaping wire coating extrusion.

Figure 8 illustrates the variation in the outer radius of the coating layer along the extrusion direction from the die exit at different braided tube haul-off speeds. Figure 8a represents the coating extrusion without a shaping section, while Figure 8b depicts the coating extrusion with a shaping section. From the figure, it is observed that the outer diameter of the coating layer either monotonically increases or decreases with changes in the traction speed of the braided pipe. Furthermore, the relationship between the shrinkage rate of the outer diameter and the traction speed does not exhibit a simple linear behavior. This stems from the fact that the coating melt is directly over the surface of the braided tube, which creates a significant drag effect when the traction speed of the braided tube exceeds the extrusion speed of the melt itself. This drag force accelerates the flow of the melt, thus changing the flow state of the melt in this direction, and this change in flow state further leads to the phenomenon of shrinkage of the outer diameter of the coating layer. On the contrary, the traction speed of the braided tube is smaller than the melt flow rate of the coating layer, and the melt is hindered by the friction of the braided tube, resulting in an increase in the outer diameter of the coating layer. As the melt gradually leaves the die in the extrusion direction, the rate of change of the outer diameter of the coating layer decreases along the distance from the extrusion direction to the die outlet, and the size gradually stabilizes after a short abrupt change. As shown in Figure 8, the difference in dimensional variation of the coating layer between the simulation of coating extrusion with a shaped section and that with an amorphous section along the extrusion direction from the muzzle die exit is less than 1%, indicating negligible change.

**Figure 9:**
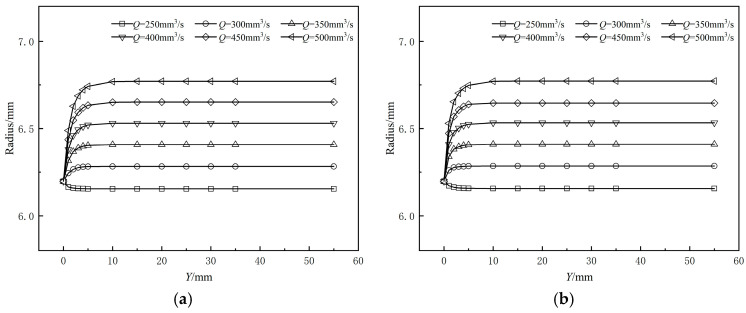
Effects of the inlet flow rate on the coating radius (**a**) without shaping section coating extrusion; (**b**) with shaping section coating extrusion.

**Figure 10:**
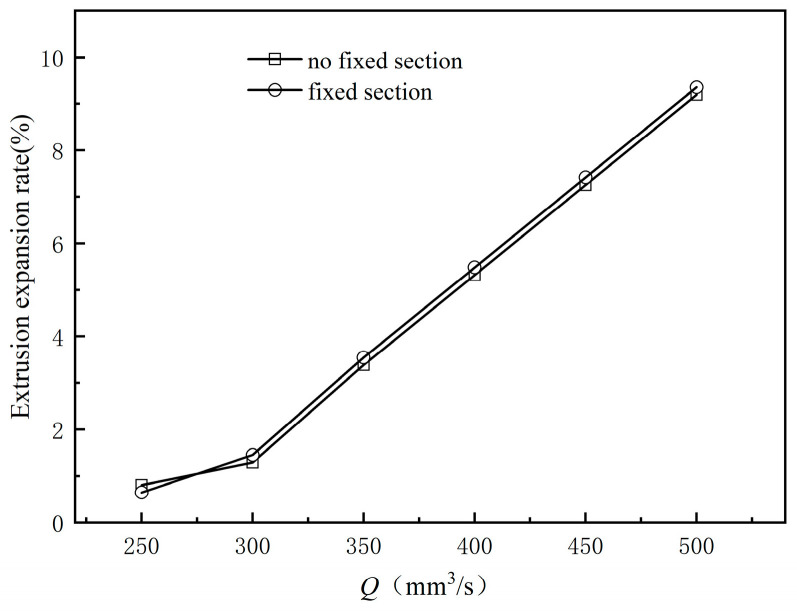
Effects of the inlet flow rate on the extrusion expansion rate.

Figure 9 shows the dimensional change of the outer radius of the coating layer along the extrusion direction from the exit of the orifice die for different inlet flow rates; Figure 10 shows the relationship between the melt inlet flow rate and the extrusion expansion rate, as shown in Equation (4):

(4) $$Extrusion\;Expansion\;Rate=\frac{R-R_{0}}{R_{0}}\times 100\%$$

Here, R and $R_{0}$ represent the radius at the end of the coating melt and the exit radius of the mouth mold, respectively. Figure 9 shows that the outer diameter of the coating layer stabilizes at a constant value after a certain distance from the melt flow exit mold. Additionally, the outer diameter increases monotonically as the melt inlet flow rate rises. This is due to the fact that as the melt inlet flow rate increases, the pressure drop across the die increases, causing the melt to expand more as it leaves the die. Figure 10 reveals that regardless of whether a shaping section is set, the extrusion expansion rate of the coating layer increases as the inlet flow rate rises. The trend and magnitude of the increase remain essentially the same. Thus, the presence of a shaping section does not mitigate the extrusion expansion phenomenon during coating extrusion.

**Figure 11:**
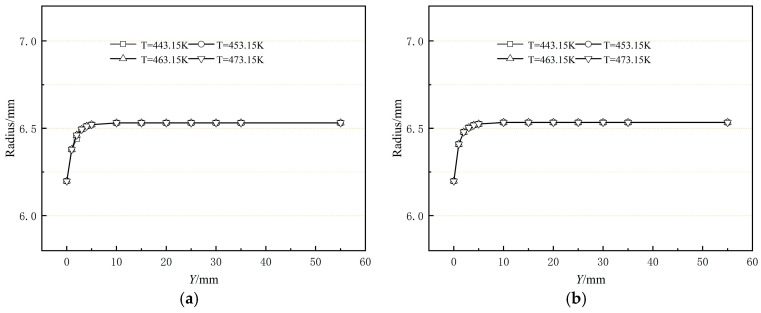
Effects of the die temperature on the coating radius (**a**) without shaping section coating extrusion; (**b**) with shaping section coating extrusion.

Figure 11 shows the dimensional change of the outer radius of the coating from the exit of the die in the extrusion direction for different die wall temperatures. The figure shows that as the process temperature rises, the outer diameter of the coating layer remains nearly constant. This behavior differs from conventional extrusion, where an increase in temperature reduces melt viscosity and enhances polymer melt fluidity, typically leading to a smaller outer diameter. In coating extrusion, however, the melt at the die exit experiences a dragging effect from the pipe, counteracting this reduction.

**Figure 12:**
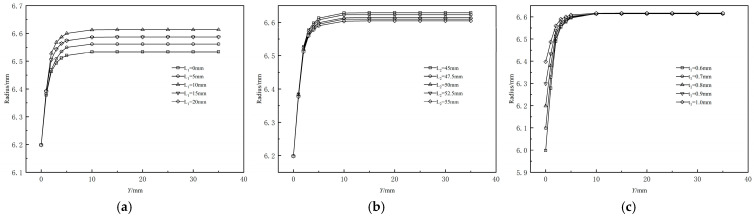
Effects of the runner parameters on the coating radius. (**a**) Effects of the length of shaping section on the coating radius; (**b**) Effects of the length of compressed section on the coating radius; (**c**) Effects of the coating thickness on the coating radius.

Figure 12a illustrates the dimensional variations in the outer radius of the cladding layer from the die exit along the extrusion direction under different shaping section lengths. A comparison reveals that the presence of a shaping section does not reduce or eliminate extrusion expansion during the coating extrusion process. Figure 12a demonstrates that as the length of the shaping section increases, the outer diameter of the extruded coating layer first increases and then decreases. When the shaping section length reaches 15 mm, further length increases have no impact on the extrusion expansion rate of the coating layer. Comparing results for different shaping section lengths, the extrusion expansion rate is lowest, approximately 0.05%, when the shaping section length is 0–5 mm.

**Figure 13:**
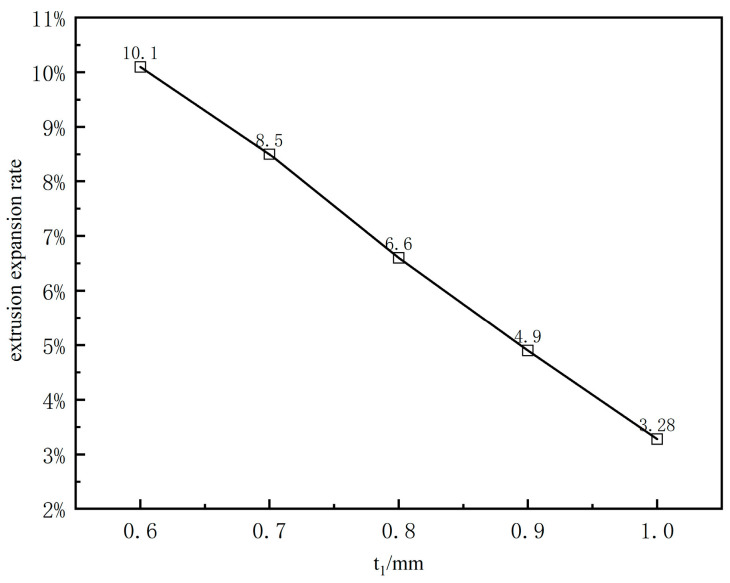
Effects of the coating thickness on the extrusion expansion rate.

Figure 12b presents the calculated results under five working conditions, where only the length of the compression section varies, while other conditions remain constant. The figure shows that as the length of the compression section increases, the stabilized outer diameter of the extruded coating layer gradually decreases. However, the reduction is minimal, indicating that changes in the compression section length have a limited effect on the extrusion expansion rate of the coating layer. Figure 12c illustrates the variation in the outer diameter of the coating layer from the die exit along the extrusion direction for different coating thicknesses. It shows that the maximum radius at the end of the extruded coating layer melt remains constant despite increased coating thickness, indicating no change in the radius at the end due to thickness variation. Figure 13 reveals that the extrusion expansion rate of the coating layer decreases as the coating thickness increases, exhibiting a monotonically decreasing trend.

### 3.3. Stability Analysis of Coating Flow Field

**Figure 14:**
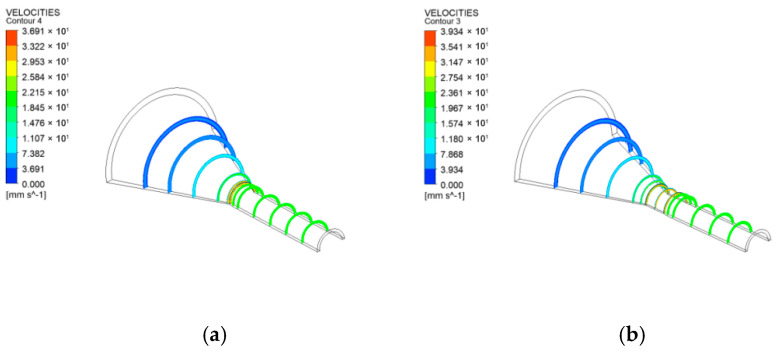
Distribution of cloud charts of velocity in different cross-section (**a**) without shaping section coating extrusion; (**b**) with shaping section coating extrusion.

To better analyze the stability of the flow field at the mouth mold exit and visually examine velocity changes across different parts of the cross-section during the coating process, velocity distributions at several cross-sections along the y-direction of the melt extrusion were compared, as shown in Figure 14.

The velocity distributions across various cross-sections in the figure indicate that velocity changes occur in two stages: inside the orifice mold (compression and sizing segments) and outside the orifice mold (free segments). Inside the coating die, the melt velocity is not fully developed; flow rates are relatively low near the die’s inner wall and the mandrel’s outer wall. However, in the central region of the annular flow channel within the compression section, the melt flow rate increases significantly, reaching its maximum near the outlet of the coating die. The velocity distribution change near the exit of the mouth mold is particularly pronounced, indicating that the melt undergoes redistribution and adjustment of flow velocity after exiting the mold. The consistent color of the velocity cloud after extrusion suggests that the melt stabilizes shortly after the initial sudden change in velocity, with the cross-sectional size gradually becoming stable as well.

To analyze the stability of the subsequent free-section flow field, this paper defines the mean value of the velocity distribution across the melt-free section and the standard deviation of the average velocity in each cross-section of the free section, as shown in Equations (5) and (6).

(5) $$\bar{V}=\frac{\sum_{i=1}^{s}V_{i}}{s}$$

(6) $$\sigma =\sqrt{\frac{\sum_{i=1}^{n}{\left({\bar{V}}_{i}-\bar{\bar{V}}\right)}^{2}}{n}}$$

where s is the number of cross-sectional velocity counting points; $V_{i}$ is the velocity at each counting point; n is the number of cross-sections; and $\bar{\bar{V}}$ is the mean value of the average velocity of each cross-section.

**Figure 15:**
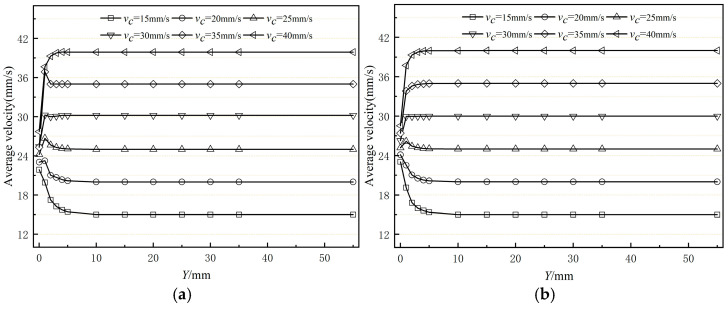
Variation in average velocity of the polymer melt along the flow direction (**a**) without shaping section coating extrusion; (**b**) with shaping section coating extrusion.

**Figure 16:**
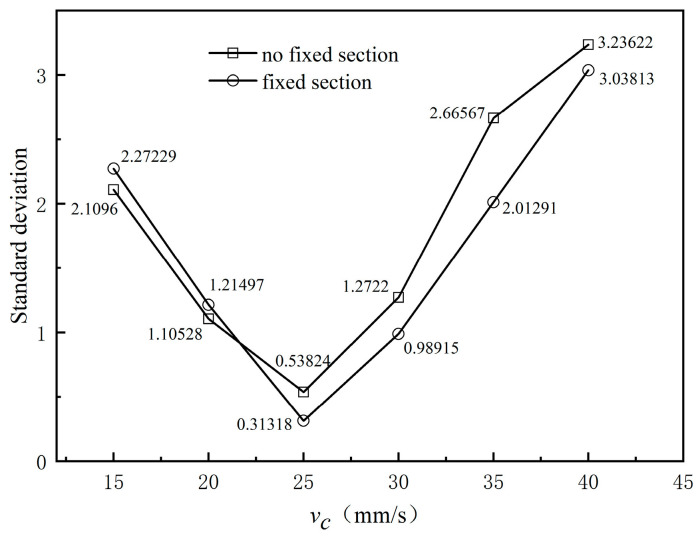
Standard deviation of different velocities.

Figure 15 presents the variation curves of the average cross-sectional speed from the exit of the compression section along the extrusion direction under different traction speeds. The figure shows that the fluctuation range of the average cross-sectional speed aligns with the fluctuation range of the coating layer’s outer diameter. Additionally, the average cross-sectional speed at the exit indicates that adjusting the haul-off speed of the braided tube has minimal effect on increasing the melt extrusion speed at the orifice die exit. Figure 16 illustrates the standard deviation of the average cross-sectional velocity at various traction speeds. The figure indicates that both the shaped and unshaped sections exhibit the same trend in velocity standard deviation. As the traction speed of the braided tube increases, the standard deviation of cross-sectional velocity initially decreases and then increases. Notably, the standard deviation is larger in the 15–20 mm/s and 35–40 mm/s traction speed intervals. This occurs because when the traction speed is either too low or too high, the melt is either dragged or hindered by the braided pipe, causing greater velocity fluctuations. In the 20 to 30 mm/s interval, the melt flow rate aligns well with the braided pipe traction speed, resulting in a small standard deviation of the average cross-sectional velocity. It is evident that if the traction speed is too low or too high, it increases the melt flow rate fluctuations, leading to size variations, uneven thickness, and poor surface quality, ultimately failing to meet product quality requirements.

**Figure 17:**
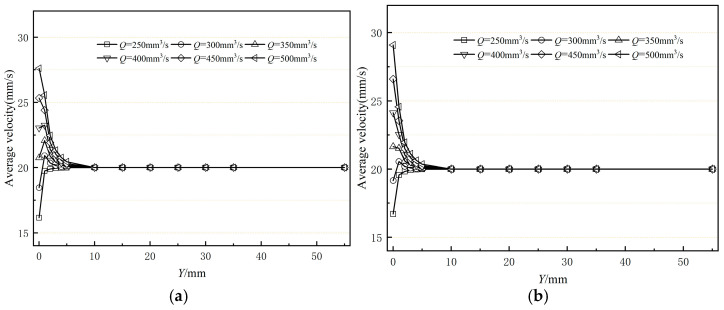
Variation in average velocity of the polymer melt along the flow direction (**a**) without shaping section coating extrusion; (**b**) with shaping section coating extrusion.

**Figure 18:**
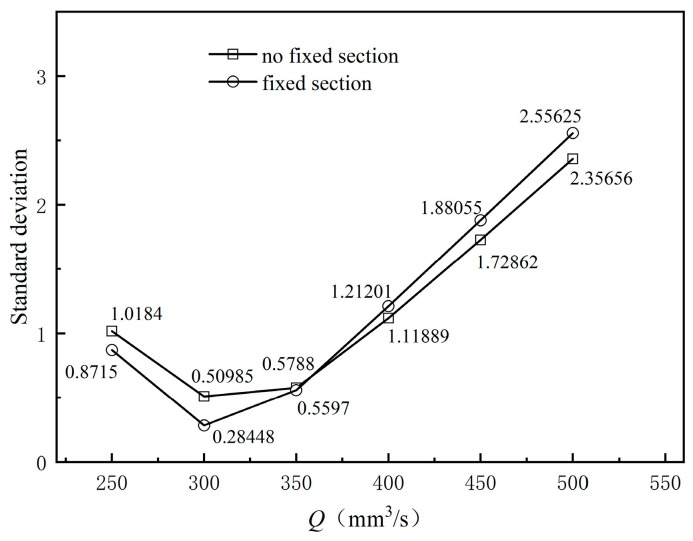
Standard deviation of different inlet flow rates.

Figure 17 illustrates the impact of melt inlet flow rate on the stability of the flow field at the orifice mold’s exit section. Analysis of Figure 17 reveals that as the melt inlet flow rate increases, the average flow velocity at the exit cross-section significantly rises. However, this increase is not strictly linear with respect to the melt inlet flow rate. This nonlinear variation is due to the complexity of the melt’s rheological properties, which affect the coating layer size through a corresponding increase in the outer diameter, causing the coating layer size change to not fully synchronize with the melt inlet flow rate change. Because the braided tube’s traction speed is constant, the cross-sectional velocity briefly changes after exiting the mouth mold before gradually converging to a consistent velocity value. Figure 18 presents the standard deviation of the cross-sectional mean velocity at various inlet flow rates. It can be observed that as the inlet flow rate gradually increases, the standard deviation of the cross-sectional mean velocity initially decreases and then increases. The standard deviation of the cross-sectional mean velocity is larger in the inlet flow rate intervals of 250–300 mm^3^/s and 350–500 mm^3^/s. This occurs due to a mismatch between the melt flow rate of the coating and the braided pipe’s traction speed. The smallest standard deviation occurs when the inlet flow rate is 300–350 mm^3^/s, indicating that at this rate, the melt flow rate aligns with the traction speed, resulting in minimal flow rate fluctuations and optimal flow field stability.

**Figure 19:**
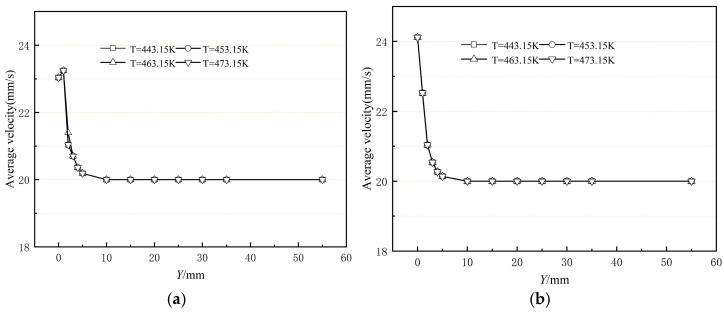
Variation in average velocity of the polymer melt along the flow direction (**a**) without shaping section coating extrusion; (**b**) with shaping section coating extrusion.

Figure 19 illustrates the variation curves of the cross-sectional average velocity from the exit of the compression section of the die along the extrusion direction at different die wall temperatures. It can be observed that changes in the exit die wall temperature do not affect the cross-sectional average velocity. Comparing Figure 19a and Figure 19b, it is evident that the average velocity fluctuation of the extruded melt at the die outlet is smaller, and the flow field is better stabilized under the current process parameter settings for the extrusion without a shaping section compared to the extrusion with a shaping section.

**Figure 20:**
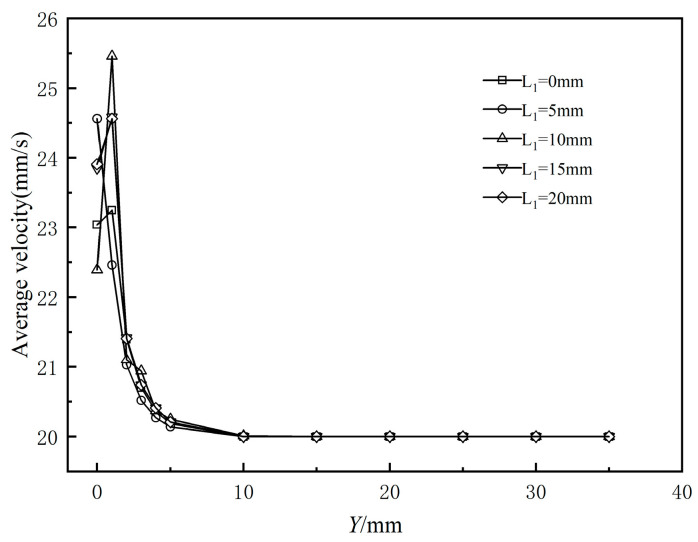
Variation in average velocity of the polymer melt along the flow direction.

**Figure 21:**
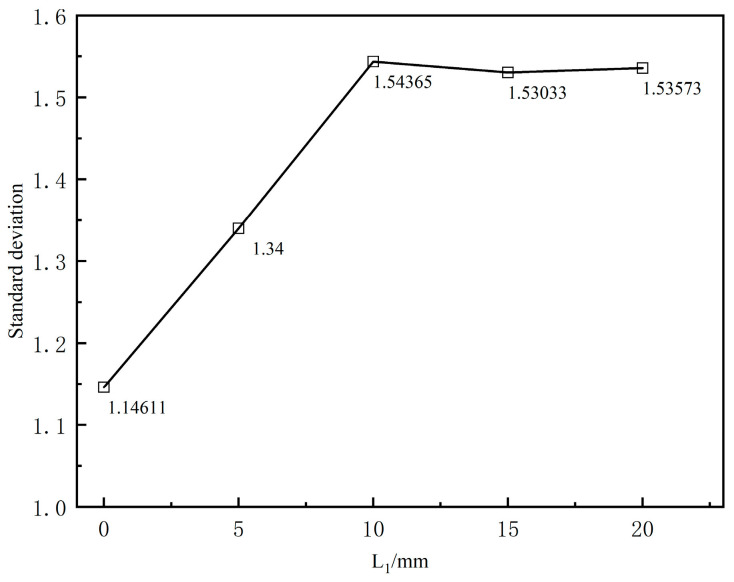
Standard deviation of different lengths of shaping section.

Figure 20 illustrates the effect of the sizing section length on the flow field stability at the die outlet cross-section. The figure shows that the fluctuation interval of the cross-sectional average velocity is primarily within 5 mm from the die exit along the extrusion direction, after which it gradually stabilizes. When the shaping section length is between 0 and 10 mm, the fluctuation of the cross-sectional average velocity in the extrusion direction increases as the shaping section length increases. Figure 21 displays the standard deviation of the cross-sectional average velocity for various shaping section lengths. It can be observed that when the shaping section length is set to 0, the standard deviation is the smallest, indicating minimal surface fluctuation of the melt flow rate and optimal flow field stability, contrasting with traditional extrusion. As the shaping section lengthens in conventional extrusion, the melt relaxes more fully while flowing through, resulting in improved stability as it exits the die. This occurs because, during wrap coating extrusion, regardless of whether a shaping section is present, the melt is dragged by the braided tube at the mouth die exit. This dragging causes velocity rearrangement, affecting the flow field’s stability.

**Figure 22:**
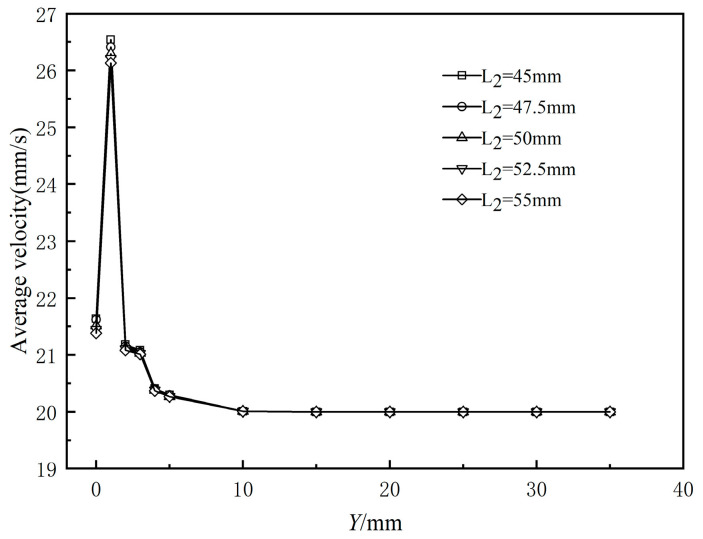
Variation in average velocity of the polymer melt along the flow direction.

The compression section length is crucial in the structural design of the wrapping head, directly impacting product quality. An appropriate compression section length ensures steady melt flow at the die outlet, reduces extrusion expansion, and enhances the cladding layer’s performance. Figure 22 presents the variation curves of the cross-sectional average speed from the die exit along the extrusion direction for different compression section lengths. It shows that the fluctuation interval of the cross-sectional average speed aligns with that of the coating layer’s outer diameter, and that changes in compression section length do not significantly affect the melt’s cross-sectional average speed.

**Figure 23:**
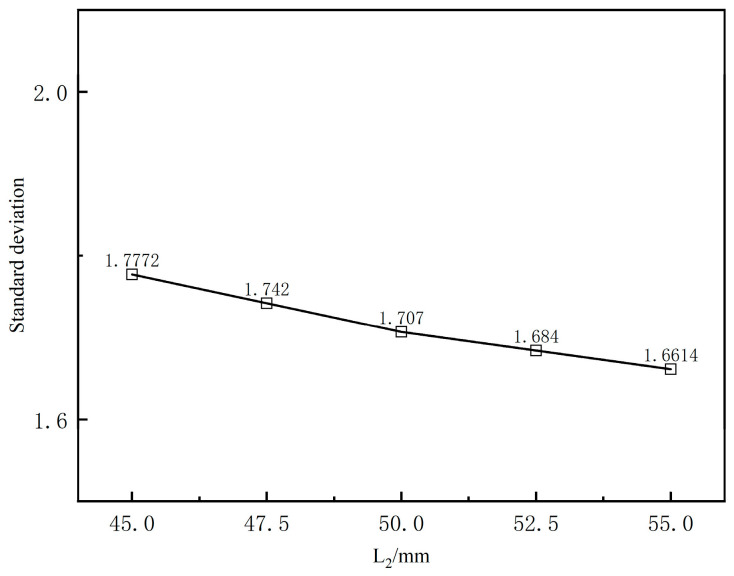
Standard deviation of different length of compressed section.

Figure 23 illustrates the standard deviation of the cross-sectional mean velocity for various compression section lengths. It can be observed that the standard deviation decreases monotonically as the compression section length increases, but the reduction is limited. This indicates that changes in compression section length have a minimal effect on flow field stability.

**Figure 24:**
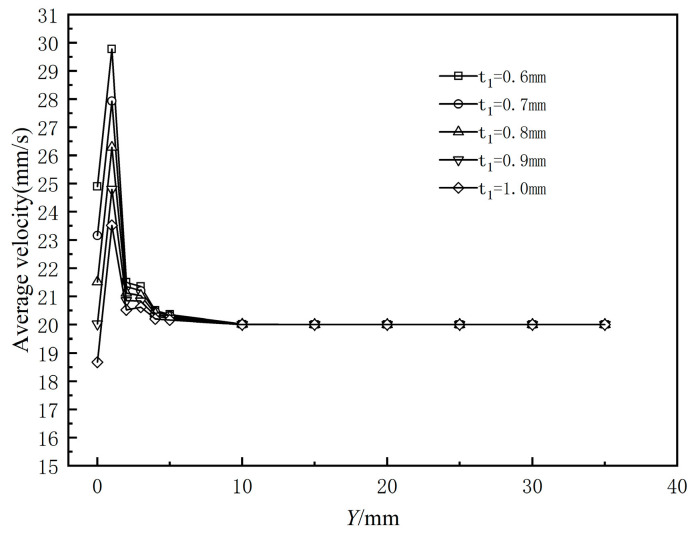
Variation in average velocity of the polymer melt along the flow direction.

**Figure 25:**
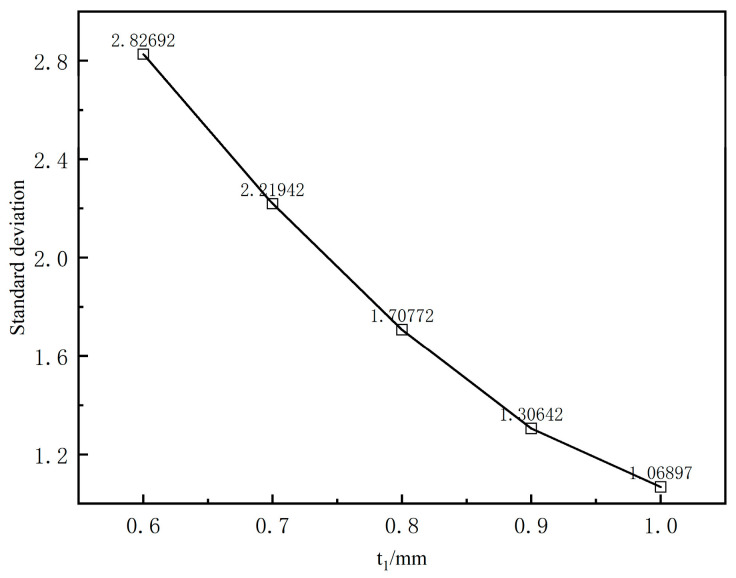
Standard deviation of different coating thicknesses.

Figure 24 presents the variation curves of cross-sectional mean velocity from the die exit along the extrusion direction for various coating thicknesses. It can be observed that the cross-sectional mean velocity exhibits a nonlinear trend, first increasing and then decreasing along the extrusion direction. Additionally, the exit cross-sectional mean velocity decreases as the coating thickness increases across the five different thicknesses. Figure 25 illustrates the standard deviation of the cross-sectional average velocity for different coating thicknesses. It can be observed that the standard deviation decreases as coating thickness increases, indicating a monotonically decreasing trend. This suggests that thinner coating thicknesses lead to greater fluctuations in melt flow velocity from the outlet along the extrusion direction, resulting in poorer flow field stability.

### 3.4. Pressure Drop Analysis

**Figure 26:**
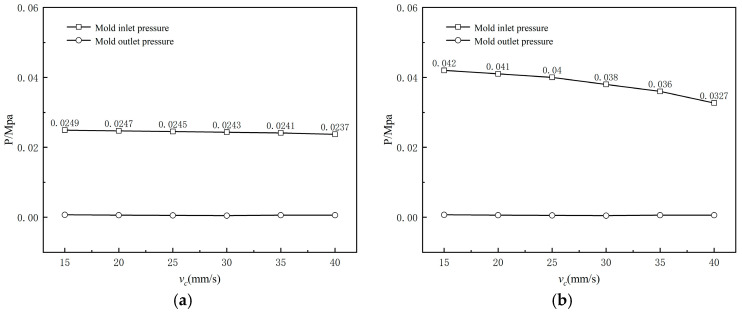
Variation of the melt pressure with varying drag speeds (**a**) without shaping section coating extrusion; (**b**) with shaping section coating extrusion.

Figure 26 depicts the variation in inlet pressure of the mouth mold at different haul-off speeds. It can be observed that as the haul-off speed of the braided tube increases, the inlet pressure of the cladding mouth mold gradually decreases, albeit with a limited reduction. Comparing Figure 26a and Figure 26b, it is evident that as the haul-off speed increases, the rate of decrease in die inlet pressure for the qualitative section is greater than that for the amorphous section. Additionally, the die pressure drop in the amorphous section-wrapped extrusion is about 40% less than that in the qualitative-section wrapped extrusion, indicating that without the shaping section, wrapped extrusion can effectively reduce the die pressure drop.

**Figure 27:**
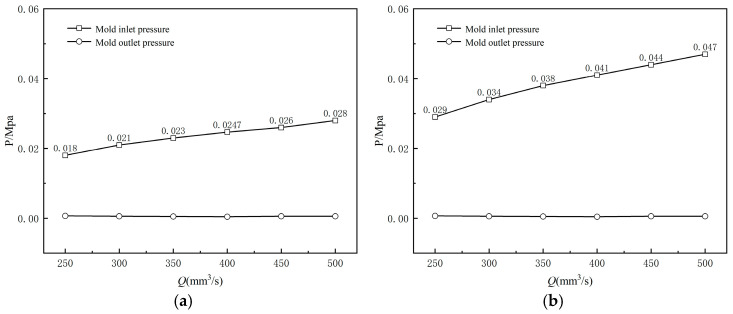
Variation of the melt pressure with varying inlet flow rate (**a**) without shaping section coating extrusion; (**b**) with shaping section coating extrusion.

Figure 27 illustrates the impact of inlet flow rate changes on the inlet pressure of the orifice mold. It can be observed that as the melt inlet flow rate increases, the screw’s rotational speed also increases, significantly enhancing the shear effect on the melt during processing. This enhanced shear effect weakens the intermolecular interaction forces within the melt, resulting in a decrease in melt viscosity. Consequently, an increase in the inlet pressure of the compression section is observed. This higher inlet pressure leads to an increase in the elastic energy stored in the melt as it flows through the compression section, enhancing its tendency to deform upon reaching the exit of the mouth mold. This is manifested macroscopically as an increase in the extrusion expansion rate of the cladding layer. Comparing the two graphs in Figure 27a and Figure 27b regarding the variation in inlet pressure of the orifice die, it can be seen that the pressure drop at the outlet die for amorphous section wrapped extrusion is approximately 35% to 40% less than that for shaped section wrapped extrusion.

**Figure 28:**
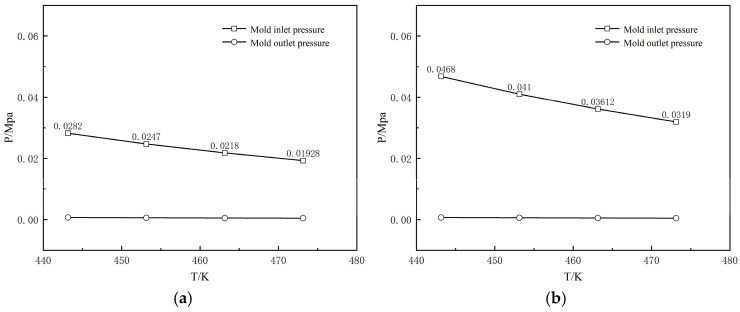
Variation of the melt pressure with varying die temperature (**a**) without shaping section coating extrusion; (**b**) with shaping section coating extrusion.

Figure 28 illustrates the effect of die wall temperature changes on the inlet pressure of the die. It can be observed that as the die temperature increases, the melt pressure within the die gradually decreases. The melt pressure at the die inlet varies with different temperatures, but the pressure at the die outlet is almost reduced to zero. Comparing Figure 28a and Figure 28b, the pressure drop at the die outlet for amorphous section wrapped extrusion is approximately 39% less than that for shaped section wrapped extrusion.

**Figure 29:**
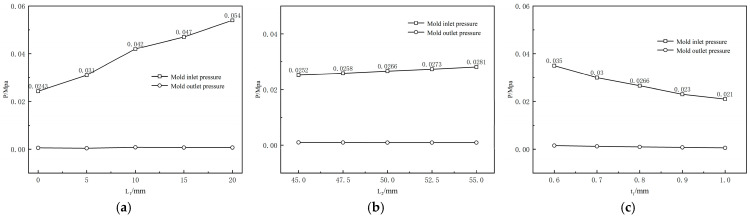
Variation of the melt pressure. (**a**) Variation of the melt pressure with varying length of shaping section; (**b**) Variation of the melt pressure with varying length of compression section; (**c**) Variation of the melt pressure with varying length of shaping section.

Figure 29a illustrates the variation in pressure drop of the mouth mold with different shaping section lengths. It can be observed that as the length of the shaping section increases, the inlet pressure of the wrapped mouth mold gradually becomes larger. Figure 29b shows the variation in inlet pressure of the mold with different compression section lengths. It is evident that as the length of the compression section increases, the inlet pressure of the mold gradually increases.

Figure 29c illustrates the variation in inlet pressure of the mouth mold with different cladding thicknesses. It can be observed that the inlet pressure of the melt in the runner decreases monotonically as the cladding thickness increases. Additionally, the rate of decrease shifts from large to small as the cladding thickness increases. The phenomenon shown in Figure 13 can be attributed to changes in melt inlet pressure. As the cladding thickness increases, the melt inlet pressure decreases. This lower inlet pressure reduces the elastic energy stored in the melt as it flows through the compression section. Consequently, the deformation tendency of the melt upon reaching the exit is weaker, which is manifested macroscopically as a reduction in the extrusion expansion rate of the cladding layer.

### 3.5. Section Design and Manufacture of the Die

**Figure 30:**
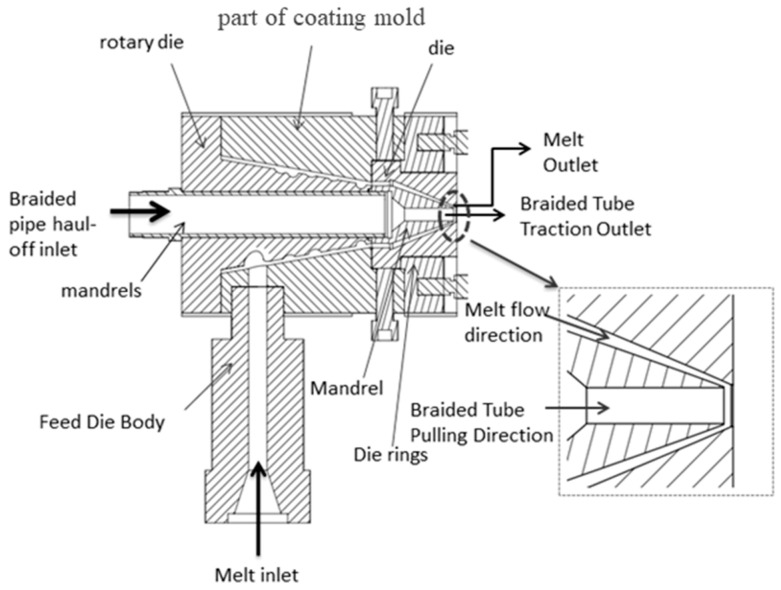
The 2D structure of the coating extrusion die.

In the coating extrusion mold design of this study, the main flow channel is formed by inserting the cone part of the diverter die into the conical groove of the outer die body. The conical flow channel, connected to the main flow channel, is created by inserting the mandrel into the conical hole of the mouth die. The absence of a flat pressure section in both the main and conical flow channels effectively reduces mold pressure, prevents material melt backflow, and ensures stable pipe production. The existing extrusion cladding dies, including the core die and mouth die, have a flow channel with a flat section. This flat section has a small cross-sectional gap area, leading to high pressure on the material melt, which causes significant machine overload, high die pressure, material melt backflow, and instability in the pipe production process. The schematic diagram of the designed structure assembly is shown in Figure 30.

**Figure 31:**
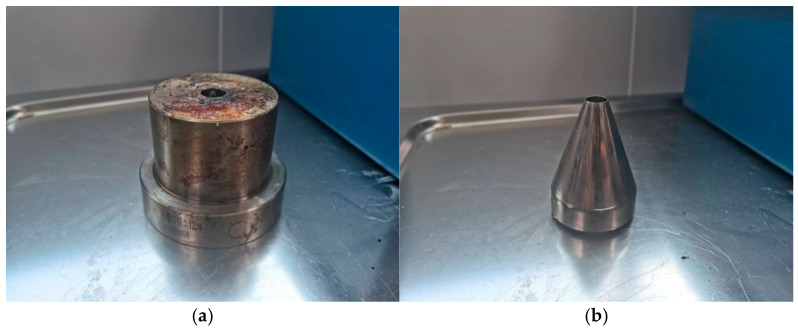

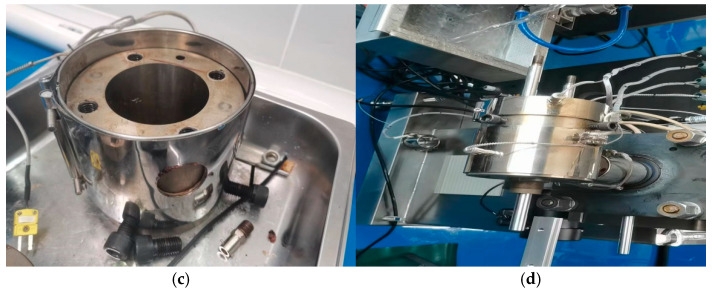
Wrap-around extrusion die: (**a**) die; (**b**) mandrel; (**c**) part of coating mold; (**d**) coating mold assemblies.

Based on the numerical simulation results, appropriate flow channel parameters were selected to complete the design of the coating extrusion mold. A CNC lathe and EDM machine were used for parts processing, and the components were then assembled to create the extrusion outlet prototype. The physical prototype is shown in Figure 31.

## 4. Coating Extrusion Experiments

### 4.1. Experimental Material and Method

**Figure 32:**
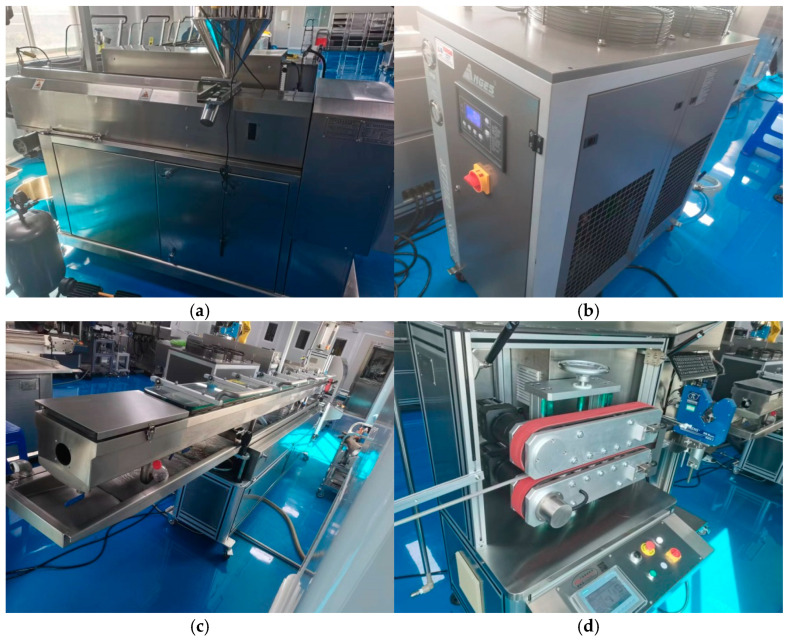
Experimental equipment of coating extrusion: (**a**) single-screw precision extruder; (**b**) freezer compartment; (**c**) cooling water tank; (**d**) servo traction machine.

The experimental material chosen is medical-grade thermoplastic polyurethane (TPU). The equipment used in the braided tube covering layer extrusion molding experiments includes a single-screw precision extruder (HRJSJ-50, 50, 28:1), precision covering extruder head molds, a cooling calibrating sink, 500/60 servo haul-offs, double-disk winders, a dual-axis online laser sizer, and melt temperature and pressure sensors, among others, as shown in Figure 32. The main machine’s control panel features eight temperature control zones. Zones one to four are designated as the screw-heating zones, zone five is the flange-heating zone, zone six is the head-heating zone, and zones seven and eight are the mold-heating zones. In the braided pipe wrapping experiment, based on the characteristics of TPU, the temperature is set to 185 °C from zones one to five, 180 °C in zone six (the machine head), and 175 °C in zones seven and eight.

### 4.2. Setting of Experimental Parameters

A stable cladding extrusion process requires the synergistic cooperation of various equipment and process parameters. The main parameters include extrusion die runner parameters, melt temperature, haul-off speed, melt inlet flow rate, and gas injection pressure. The cladding extrusion die runner parameters are fixed upon the completion of the extrusion die, while the remaining parameters can be adjusted within the basic equipment parameter range. These adjustable parameters can be further subdivided into two categories: directly adjustable parameters and indirectly adjustable parameters.

Melt temperature control: The heating of the melt relies on stainless steel heating collars placed on the outer surfaces of the extruder die and barrel. These collars can quickly heat the extruder barrel and die up to 300 °C and maintain stable temperatures. The experiment showed that the thermoplastic polyurethane melt exhibited good fluidity between 170 °C and 220 °C without significant degradation. Consequently, the extrusion system can sustain the process temperature within this range for extended periods when wrapping the braided tube.

Melt inlet flow rate control: The extruder used in the experiment is equipped with a single screw featuring an L/D ratio of 28 and a diameter of 50 mm. The screw’s rotational speed can be directly controlled via the console. Due to the one-to-one relationship between the screw rotational speed and the melt inlet flow rate, this chapter uses the screw rotational speed to represent changes in the melt inlet flow rate. This approach simplifies the experimental results and facilitates discussion.

Control of traction speed: The servo traction machine used in this experiment features a Siemens human–machine interface on its control panel, allowing direct control of the traction speed.

### 4.3. Experimental Methodology Steps

**Figure 33:**
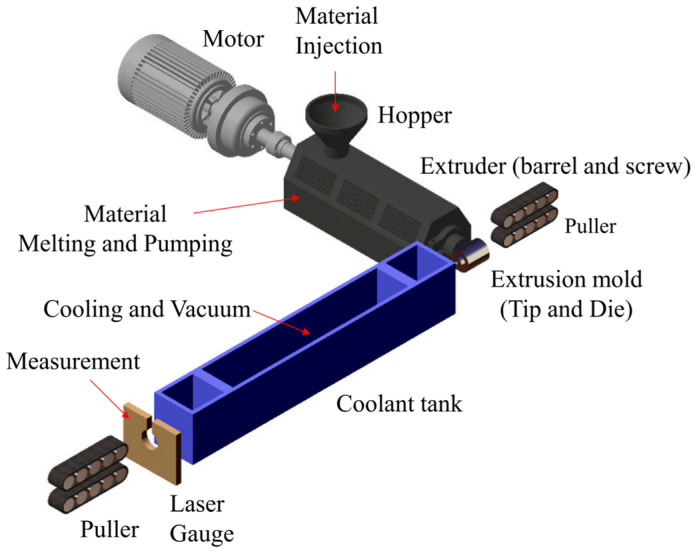
The coating-extrusion system for composite medical tubing.

The overall process, including all the experiments, is illustrated in Figure 33. The main process and steps for the extrusion of the braided pipe cladding layer include the following: (1) Install the head of the cladding extruder and complete the connection of pipeline routes in various locations. (2) Add dried TPU pellets to the extruder hopper. (3) Turn on the power supply for the various devices in the cladding extrusion system and set the parameters according to processing requirements. (4) Observe whether the material from the outlet of the mouth die is centered correctly; if not, adjust the mouth die to ensure uniform wall thickness. (5) Once the temperature at each location reaches the set value, pass the braided tube through the mouth die from the head haul-off machine, through the cooling water tank, and finally to the tail haul-off machine. (6) Press the extruder’s discharge switch to conduct the coating experiment and verify the usability of the coating extrusion outlet mold. In practice, the heated braided tube enters the rear of the mold, which is typically maintained at the same temperature as the melt inside the mold. Simultaneously, the polymer melt is extruded from the mold and thermally bonded to the braided tube that passes through the center of the mandrel, facilitating the continuous extrusion of the coating layer.

### 4.4. Experimental Results

**Figure 34:**
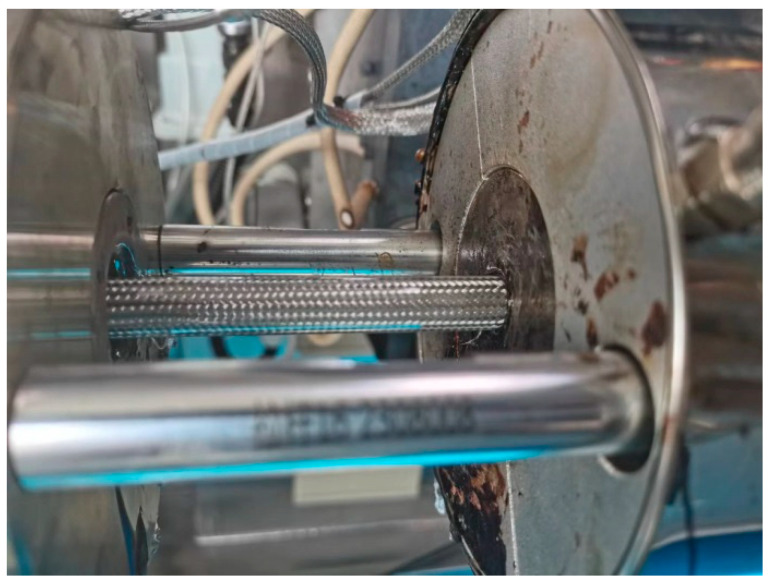
Stabilized coating extrusion.

Using the designed extrusion die for the cladding layer, a typical experimental photograph obtained after continuous process parameter adjustments is shown in Figure 34. During this time, the extruder screw speed was set to 10 r/min, the haul-off speed was 2.0 m/min, and the temperature of the extrusion die was 180 °C. From the figure, it can be seen that, at this time, the extruded products have a uniform size of the wrapping layer and smooth surface, indicating that the extrusion process is stable, and the effect and quality of the extruded conduit is more ideal.

## 5. Conclusions

This study employed numerical simulations to investigate the impact of primary process parameters and flow channel parameters on the characteristics of overlay extrusion of medical thin-walled tubing. Based on these simulation results, an over-molding extrusion die specifically for medical thin-walled tubing was designed. The validity of the mold design was confirmed through over-molding extrusion experiments, resulting in the following conclusions:

1. Whether or not the shaping section of coating extrusion is set up, the size of the coating layer and the homogeneity of the flow field in coating extrusion exhibit similar changes in response to variations in process parameters such as braided pipe haul-off speed, melt inlet flow rate, and mouth mold temperature. Unlike previous studies on conventional extrusion, the extrusion expansion in coating extrusion with a shaping section is almost identical in appearance to that without a shaping section. In coating extrusion, the presence of a shaping section does not reduce or eliminate the extrusion expansion.
2. Compared with the coating extrusion with a shaping section, the coating extrusion without a shaping section can reduce the pressure drop of the die and reduce energy consumption. In actual production, due to the thin-wall requirement of the coating layer, the core die and the mouth die formed by the cross-section of the straight section of the flow channel gap area are small and the material melt in the shaping section of the pressure is too large, resulting in a large extruder overload, high die pressure, and material melt reflux, so the amorphous section of the package extrusion has more advantages than the qualitative section of the package extrusion.

This study enhances the precision of the coating layer in the coating extrusion process, which is actively applied in the field of minimally invasive and interventional procedures. It also enriches the theory of cladding extrusion preparation for thin-walled tubes, providing a reference for formulating process parameters in actual production. It is also very helpful in designing coating extrusion molds, which can be difficult to predict.

## Figures and Tables

**Table 1:** Mechanical properties of TPU ESTANE-58271(Wycliffe, OH, USA).

Material	Manufacturer	Hardness	Specific Gravity	Tensile Strength	Melting Point
TPU	Lubrizol	85 [A]	1.21 [g/cm^3^]	51.7 [Mpa]	120 [°C]

**Table 2:** Properties of TPU ESTANE-58271 for numerical analysis.

Parameters	Value
$\eta_{0}$	6295
λ	0.094
n	0.389
$\eta_{\infty }$	6.3 × 10^−4^
α	7176
$T_{a}$	180
$T_{0}$	−273.15

**Table 3:** Model size of the coating extrusion.

Unit	L_1_	L_2_	L_3_
mm	50	10	45

**Table 4:** Coating extrusion processing and process variables.

Research Objective	Invariant Variable	Variables
Drag velocity	$Q=400\;{mm}^{3}/s$; T=453.15 K	$v_{c}$
inlet flow rate	$v_{c}=20\;mm/s$; T=453.15 K	Q
die temperature	$v_{c}=20\;mm/s$; $Q=400{mm}^{3}/s$	T
Length of shaping section	$L_{2}=50\;mm$; $t_{1}$ = 0.8 mm	$L_{1}$
Length of compressed section	$L_{1}=0\;mm$; $t_{1}$ = 0.8 mm	$L_{2}$
Coating thickness	$L_{1}=0\;mm$; $L_{2}=50\;mm$	$t_{1}$

## Data Availability

The original contributions presented in the study are included in the article; further inquiries can be directed to the corresponding author.
